# When alternatives to screening should be the priority

**DOI:** 10.1177/09691413221102322

**Published:** 2022-06-23

**Authors:** Nicholas J Wald, Stephen W Duffy, Allan Hackshaw

**Affiliations:** 1 Institute of Health Informatics, University College London, London, UK; 2 Wolfson Institute of Population Health, Queen Mary University of London, UK; 3 Cancer Institute, University College London, London, UK

Primary prevention should almost always be regarded as the preferred public health policy. But in the absence of an effective primary preventive intervention, screening for several medical disorders has (and will) become standard practice. However, better public health education and primary preventive strategies arising from significant research should replace or be used alongside screening as the main public health intervention.

The UK National Screening Committee has a set of criteria for appraising the viability, effectiveness and appropriateness of a screening programme.^1^ One of those criteria is: “All the cost-effective primary prevention interventions should have been implemented as far as practicable.” This reflects the principle that where feasible, primary prevention takes a higher priority than screening. The folic acid issue is a paradigm of this principle.

In this issue, the Special Article by Professor Wald^2^ summarises the reasons why folic acid fortification in the prevention of neural tube defects (NTDs) should be the priority and screening a back-up. In many countries, including the UK, antenatal screening for NTDs with the option of women having a termination of an affected pregnancy is standard practice even though primary prevention, through folic acid fortification of flour and grains at a level that can achieve substantial NTD prevention, is overlooked.

Accordingly, the *Journal of Medical Screening* will from now on consider publishing papers on specific primary preventive strategies that can directly prevent serious medical disorders either in tandem with screening or instead of screening. These articles can cover efficacy, cost-effectiveness and acceptability of appropriate interventions. We hope that this will extend the scope of the Journal to reflect the health benefits of primary prevention that may have been overshadowed by medical screening.
